# Chlorophyll Detection by Localized Surface Plasmon Resonance Using Functionalized Carbon Quantum Dots Triangle Ag Nanoparticles

**DOI:** 10.3390/nano12172999

**Published:** 2022-08-30

**Authors:** Nur Afifah Ahmad Nazri, Nur Hidayah Azeman, Mohd Hafiz Abu Bakar, Nadhratun Naiim Mobarak, Tg Hasnan Tg Abd Aziz, Ahmad Rifqi Md Zain, Norhana Arsad, Yunhan Luo, Ahmad Ashrif A. Bakar

**Affiliations:** 1 Department of Electrical, Electronic and Systems Engineering, Faculty of Engineering and Built Environment, Universiti Kebangsaan Malaysia, Bangi 43600, Selangor, Malaysia; 2 Department of Chemical Sciences, Faculty of Sciences and Technology, Universiti Kebangsaan Malaysia, Bangi 43600, Selangor, Malaysia; 3 Institute of Microengineering and Nanoelectronics, Universiti Kebangsaan Malaysia, Bangi 43600, Selangor, Malaysia; 4 Guangdong Provincial Key Laboratory of Optical Fibre Sensing and Communications, College of Science and Engineering, Jinan University, Guangzhou 510632, China; 5 Institut Islam Hadhari, Universiti Kebangsaan Malaysia, Bangi 43600, Selangor, Malaysia

**Keywords:** carbon quantum dots, surface plasmon resonance, silver nanoparticles, chlorophyll, optical sensor

## Abstract

An optical sensor-based localized surface plasmon resonance (LSPR) sensor was demonstrated for sensitive and selective chlorophyll detection through the integration of amino-functionalized carbon quantum dots (NCQD) and triangle silver nanoparticles (AgNPs). The additions of amino groups to the CQD enhance the detection of chlorophyll through electrostatic interactions. AgNPs-NCQD composite was fabricated on the surface of the silanized glass slide using the self-assembly technique. The experimental results showed that the AgNPs-NCQD film-based LSPR sensor detects better than AgNPs and AgNPs-CQD films with a good correlation coefficient (R^2^ = 0.9835). AgNPs-NCQD showed a high sensitivity response of 2.23 nm ppm^−1^. The detection and quantification limits of AgNPs-NCQD are 1.03 ppm and 3.40 ppm, respectively, in the range of 0.05 to 6 ppm. Throughout this study, no significant interference was observed among the other ionic species (NO_2_^−^, PO_4_^−^, NH_4_^+^, and Fe^3+^). This study demonstrates the applicability of the proposed sensor (AgNPs-NCQD) as a sensing material for chlorophyll detection in oceans.

## 1. Introduction

Coastal upwelling is where cold water pulling from a warmer bottom surface results in high levels of primary productivity and fishery production [1]. This phenomenon brings phytoplankton and nutrients from deeper ocean layers to the surface, drawing predators such as marine snails, fish, and birds [2]. Moreover, coastal ocean depletion also invites adverse effects on tourism due to algae blooming in the coastal zone. Given the importance of phytoplankton in oceans, various attempts have been made to detect chlorophyll compounds [3,4,5]. Previously, high-performance liquid chromatography, ultraviolet and visible light, and fluorometer spectroscopy were applied to determine the chlorophyll in the oceans. However, spectroscopy and chromatography have a limitation that may impose a limited sample size that is costly and labour-consuming [6]. The ocean colour remote sensor technologies are employed as one of the general methods for algal biomass detection and water quality [7,8]. Features around the sea are independently affected by water colour. For example, the absorption and scattering of total suspended solids and coloured dissolved organic matter decouples the light absorption of phytoplankton. Limitations of existing satellite sensors are also due to weather, spectral, spatial, and temporal resolution affecting colour data.

Localized surface plasmon resonance (LSPR) is an optical spectroscopic method caused by interactions of incident light with the surface of metallic structures [9]. These interactions cause the electron density to oscillate at a resonance frequency that strongly depends on the composition and geometry of nanoparticles (NPs) and the environment refractive index [10]. Gold (Au) and silver (Ag) are common noble metals among the NPs. In addition, there are various shapes of nanoparticles such as spheres, nanorods, nanoshells, and triangular. Among them, the triangular shape of AgNPs has edges and sharpness at the tips, leading to enhanced electric fields crucial for surface-enhanced spectroscopies [11]. Although AuNPs are commonly explored due to chemical stability and biocompatibility, AgNPs provide more remarkable sensitivity outcomes in sensor applications. Thus, LSPR sensors can yield great sensitivity in analytical detection. However, the nanoparticles need to be modified with a layer of suitable materials for analytic measurements at selectively low concentration levels of analytes [12,13,14].

Carbon-based nanomaterials are widely utilized in sensors design due to their unique physicochemical properties. Carbon quantum dots (CQD) are fluorescent carbon nanoparticles with a size below 10 nm. CQD consist of a carbon core with surface elements, mainly oxygen, hydrogen, and nitrogen, with different ratios depending on their synthesis routes. Functionalization or surface passivation strategies in sensor applications generally involve adding specific functional groups such as amino, hydroxyl, and carboxyl to improve polarity and chemical activity [15]. In addition, many additional materials have been used, such as ethylenediamine, chitosan, and branched polyethyleneimine. Introducing passivated CQD with polyamines that function with free amines significantly enhances the selective binding with specific ions [16]. For example, Yang et al. synthesize polyamine carbon dots using a microwave-assisted method using renewable xylan and branched polyethyleneimine as precursors [17]. The CQD polyamine act as a fluorescence probe for tannic acid detection sensitively and selectively in water and ethanol solutions. This CQD polyamine sensor was successfully applied in real sample applications; hence, it offers an excellent probe for tannic acid detection in practical applications.

This research aims to evaluate the possible detection of chlorophyll using a functionalized carbon quantum dots optical sensor-based LSPR. In this research, amino-functionalized CQD were synthesized using two steps of hydrothermal methods using polyethyleneimine precursor as the source of amino groups. The designed CQD, NCQD, and AgNPs were further characterized by high-resolution transmission element microscopy (HR-TEM) and Fourier transform infrared spectroscopy (FT-IR) to confirm the morphology and surface functional groups. The composite of functionalized CQD and triangular silver nanoparticles film was used to improve the selectivity and sensitivity of the LSPR sensor. The addition of amine groups on pristine CQD enhances the interaction of chlorophyll with the NCQD through electrostatic interactions. The wavelength shift of the LSPR spectrum was observed to characterize the sensor performance (linearity, range, sensitivity, limit of detection and quantification, selectivity) with different chlorophyll concentrations.

## 2. Materials and Methods

### 2.1. Materials

Citric acid, polyethyleneimine, silver nitrate, trisodium citrate, sodium borohydride, 3-aminopropyltrimethoxysilane (97%) (APTES), ethanol, and acetone were purchased from Sigma Aldrich. Ethylenediamine, metal salts (NaH_2_PO_4_, FeCl_3_, NaNO_3_, and NaNO_2_), and ammonia solution (30%) were purchased from R&M Chemicals, and total chlorophyll was purchased from Tokyo Chemical Industry Co., Ltd.

### 2.2. Characterizations

The characterization of synthesized CQD, NCQD, and AgNPs was determined by a Zetasizer Nano ZS dynamic light scattering (DLS) (Malvern, UK), Fourier-transform infrared (Perkin Elmer, Waltham, Massachusetts, USA), high-resolution transmission electron microscope (HR-TEM) (JEOL, Peabody, Massachusetts, USA), and photoluminescence spectrophotometer (FLS920, Edinburgh Instrument, Kirkton Campus, UK).

### 2.3. Synthesis of NCQD

**Scheme 1:**
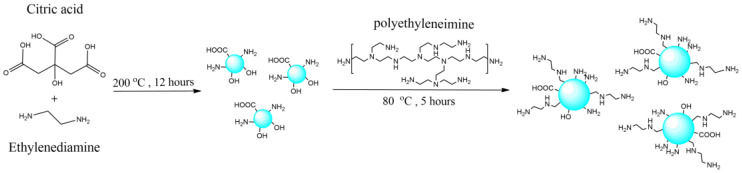
Synthesis of NCQD.

The functionalized CQD was synthesized using two steps of hydrothermal methods (Scheme 1) [18,19]. The citric acid (1.0507 g) was dissolved in 10 mL of deionized water, and 335 μL of ethylenediamine was added to the mixture and heated at 200 °C for 12 h using a Teflon-line autoclave. Upon the reaction, the CQD solution was naturally cooled at room temperature. Further, 1 mL of polyethyleneimine solution was added, and the resulting mixture was heated again for 5 h at 80 °C. The resulting solution was centrifuged at 4000 rpm for 30 min and filtered using a 0.22 µm membrane filter. Finally, the supernatant solution was collected and dialyzed using a dialysis bag for a day. The NCQD solution was obtained and kept for further characterization.

### 2.4. Preparation of AgNPs-NCQD Composite on LSPR Substrate

**Scheme 2:**
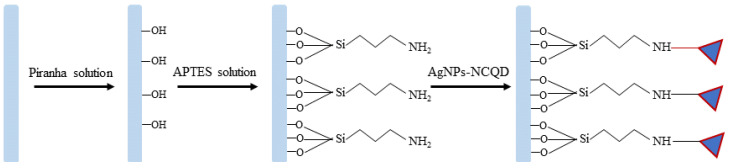
Schematic diagram for fabrication process of AgNPs-NCQD composite.

The changes in the plasmon resonance wavelength of maximum absorption or scattering are monitored as a function of changes in the chemical and physical environment of the nanoparticle surface [20]. For example, silver nanoparticles generate stronger plasmon resonances than gold nanoparticles. This may be due to the high reflectivity of metal surfaces. Moreover, silver nanoparticles also indicate numerous advantages for probes, such as higher extinction coefficients, sharper extinction bands, and high field enhancement [21]. According to the previous report, the triangular silver nanoparticles were synthesized using silver nitrate, trisodium citrate, sodium borohydride, and H_2_O_2_ by the chemical reduction process at room temperature [22]. The composite of AgNPs-NCQD (4:1) solution was grown on the glass slide using the self-assembly technique. First, the preparation of glass slides was begun by immersing in piranha solutions and the ultrasonic treatment in acetone and ethanol, respectively, for 15 min. Then, the clean glass was submerged in 5% APTES solution for 1 h and rinsed with ethanol solution. Finally, the silanized glass was immersed in the composite solution overnight and dried on the hot plate at 60 °C. g LSPR. Scheme 2 depicts the fabrication procedure of glass slides using the composite AgNPs-NCQD.

### 2.5. Detection of Chlorophyll

**Figure 1:**
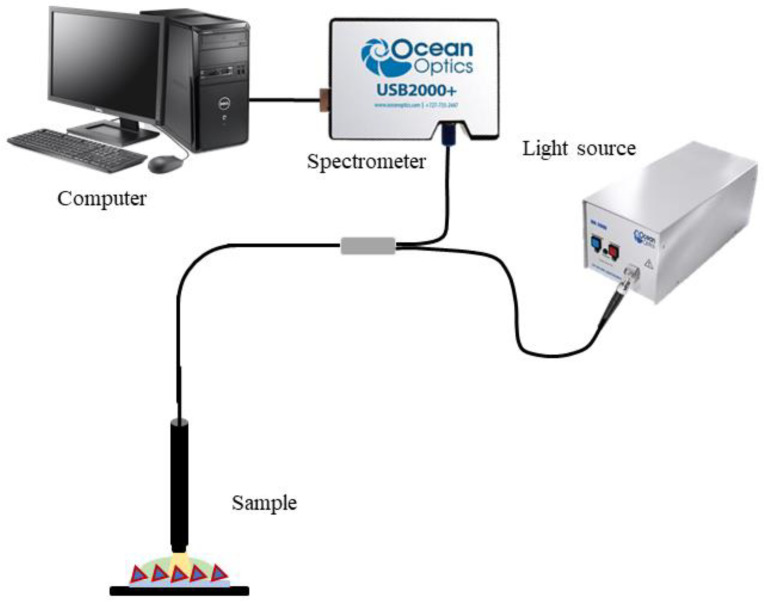
Experimental setup for detection of chlorophyll using LSPR setup.

The LSPR experimental setup consisted of a light source (DH-2000-BAL, Ocean Optic, Orlando, Florida, USA) with a spot size of 1–2 mm^2^ and an HR4000CG-UV-NIR (Ocean Optics) spectrometer fitted with a reflection probe with numerical aperture of 0.22. Reflectivity spectra of the AgNPs, AgNPs-CQD, and AgNPs-NCQD were collected across a wavelength range of 350–850 nm within a few minutes of spectral capture time. The probe was positioned to an optimal height above the sample, as shown in Figure 1.

## 3. Results and Discussions

### 3.1. Characterization of as-Synthesized CQD and AgNPs

**Figure 2:**
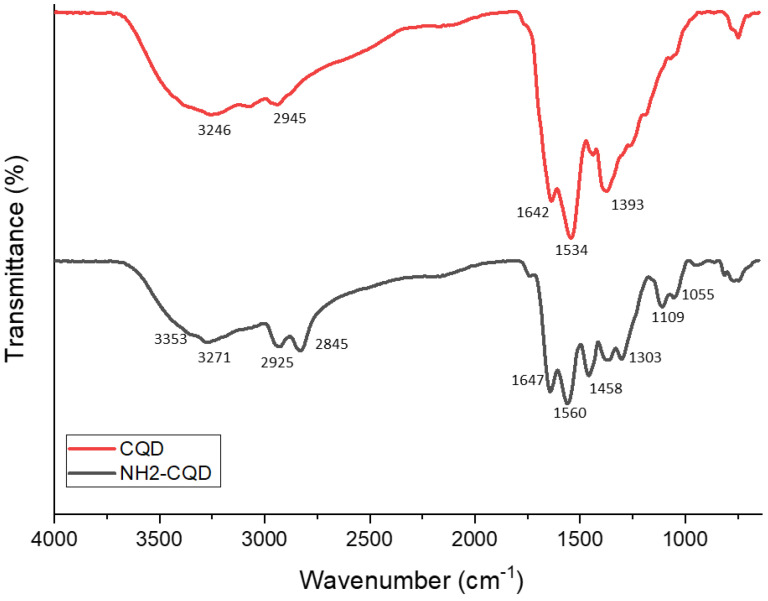
FTIR absorption of CQD (red) and NCQD (black).

The prepared CQD, amine functionalized CQD (NCQD), and AgNPs were characterized by Fourier transforms infrared (FTIR), UV-vis and photoluminescence spectrophotometer, and high-resolution transmission electron microscopy (HRTEM). FTIR spectra of CQD and NCQD are shown in Figure 2. In CQD FTIR spectra, two absorption peaks at 1534 cm^−1^ and 1642 cm^−1^ are caused by N-H bending and stretching of the amide bond. In addition, the peaks at 2945 cm^−1^ and 3246 cm^−1^ correspond to CH stretching vibration and OH/NH. For NCQD FTIR spectra, the NH peaks appear at 1363 and 1647 cm^−1^, CN stretching aromatic amine at 1303 cm^−1^, and CH at 1458 cm^−1^ and 2941 cm^−1^ [14]. In addition, the broad peak around 3271 cm^−1^ corresponded to OH stretching vibrations, whereas the peak at 3353 cm^−1^ indicated the presence of the NH group [18]. The appearance peak of the NH and CN group in FTIR spectra proved the binding of PEI on the surface of pristine CQD [23]. The measurement of zeta potential provides insight into the surface charge of NCQD. The surface charge of separate CQD and NCQD are −19.7 and 0.57 mV, respectively. The zeta potential shifts from negative to positive, indicating the presence of positively charged surroundings in the NCQD [24].

**Figure 3:**
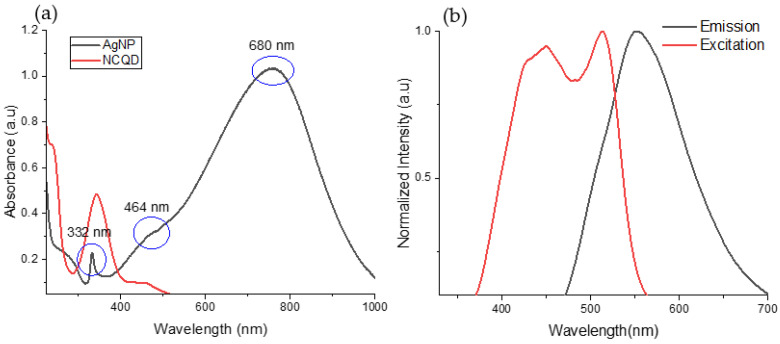
(**a**) UV-Vis absorbance and (**b**) photoluminescence excitation (red) and emission spectra (black) wavelength, respectively.

**Figure 4:**
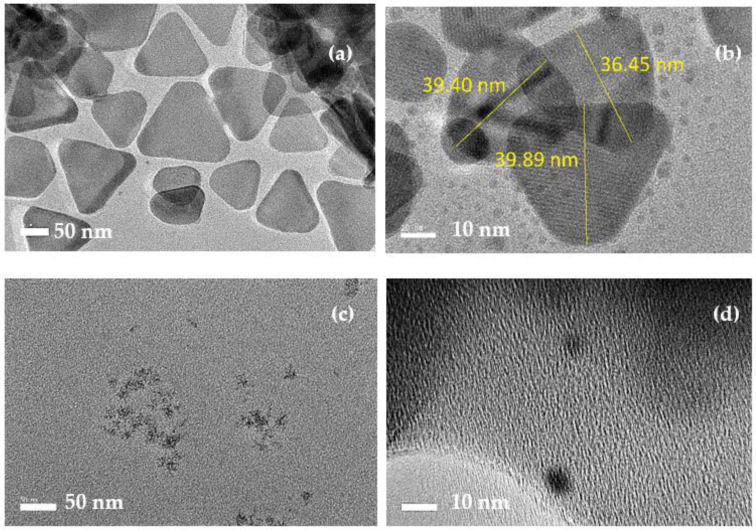
HRTEM images scale bar of 50 nm of (**a**) AgNPs and (**c**) NCQD, and scale bar of 10 nm (**b**) AgNPs and (**d**) NCQD.

Figure 3a,b represent the UV-Vis absorption and fluorescence spectra of NCQD, respectively. The NCQD showed two noticeable absorption bands at 238 and 342 nm, which were attributed to π-π * transition (C=C) and n-π * transition of (C=O), respectively [25]. The fluorescent excitation spectra of the NCQD were acquired at the maximum excitation wavelength of 512 nm and featured an optimum emission peak at 551 nm. NCQD solution showed a bright blue colour under illumination with a UV light at 360 nm. Meanwhile, the AgNP exhibited three absorption peaks. The absorption bands at 332, 464, and 680 nm correspond to out-of-plane quadrupole, in-plane quadrupole, and in-plane dipole plasmon resonances, respectively [22]. The morphologies and sizes of AgNPs and NCQD were characterized by HRTEM. As shown in Figure 4a,b, a precise triangle shape within the size ranges from 30 to 40 nm. Meanwhile, Figure 4c,d showed that NCQD exhibited a spherical shape with a size below 10 nm.

### 3.2. Sensing Performance

**Figure 5:**
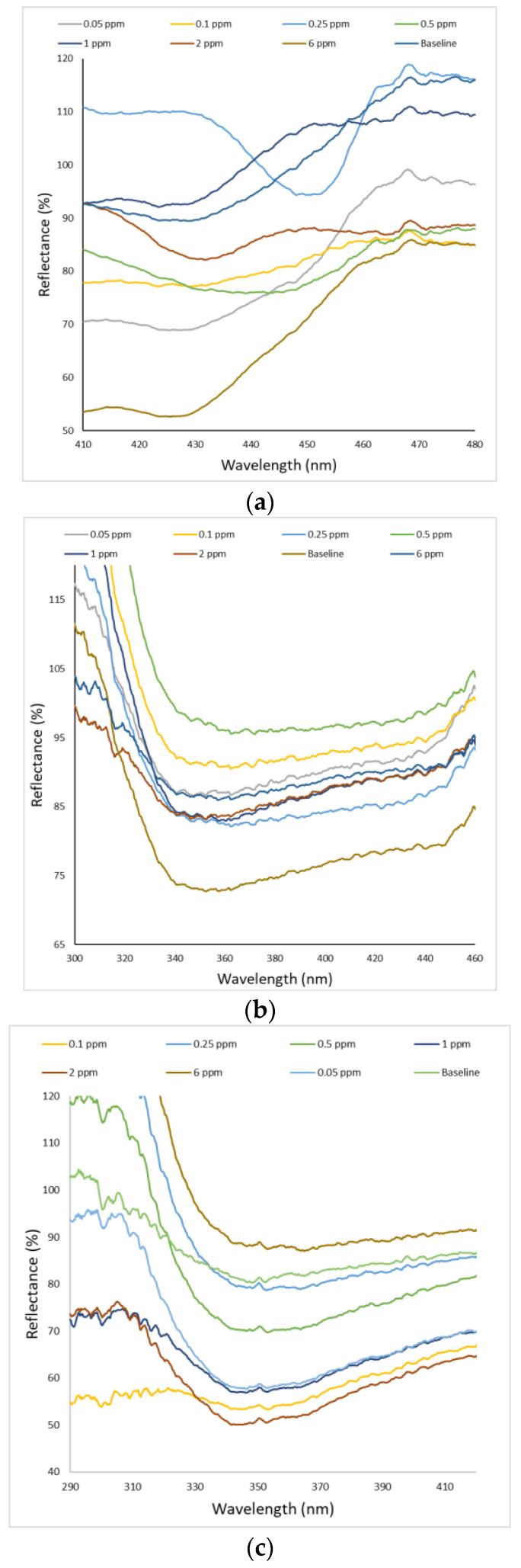
The reflectance spectra of the LSPR sensor in the presence of chlorophyll solution with different concentrations ranging from 0.05 to 6 ppm of (**a**) AgNPs, (**b**) AgNPs-CQD, and (**c**) AgNPs-NCQD.

The capability of the fabricated AgNPs-NCQD film-based LSPR sensor was investigated for the quantitative detection of chlorophyll solutions. In addition, the efficiency and sensitivity of AgNPs-NCQD were compared with AgNPs and AgNPs-CQD. This report used 90% acetone as the reference for all film sensing materials. As shown in Figure 5a, when AgNPs were exposed to chlorophyll, the wavelength value was shifted from baseline (430 nm) to 427.38, 429.74, 448.9, 439.72 m, 423.43, 429.74, and 424.22 nm for 0.05, 0.1, 0.25, 0.5, 1, 2, and 6 ppm of chlorophyll concentrations, respectively. Figure 5b depicts the spectral changes of AgNPs-CQD in response to the different concentrations of chlorophyll. The wavelength shift values change to be 4.51, 10.05, 5.82, 6.09, 5.29 m, 7.42, and 10.58 nm. Meanwhile, Figure 5c demonstrated the reflectance spectrum of AgNPs-NCQD when introduced to different chlorophyll concentrations. The initial reflectance peak of the AgNPs-NCQD baseline is at 348.48 nm. Then, after the film of AgNPs-NCQD was in contact with the 0.05 ppm chlorophyll, the reflectance peak shifted to 346.37 nm. Consequently, the shifted wavelength (Δ*ʎ*) values for 0.1, 0.25, 0.5, 1, 2, and 6 ppm chlorophyll were 2.91, 4.24, 4.77, 5.3, 6.89, and 16.42 nm, respectively. Based on the results above, it has been found that the wavelength shift of LSPR reflectance was not consistent. This behaviour is inconsistent with the basic understanding of triangular silver nanoparticles’ shape and size. Compared with spherical or quasi-spherical AgNPs, the triangular AgNPs exhibited sharp corners resulting in a redshift of reflectance peaks. However, multiple modes of triangular AgNPs can be present simultaneously, which leads to the less pronounced redshift reflectance peaks [26].

**Figure 6:**
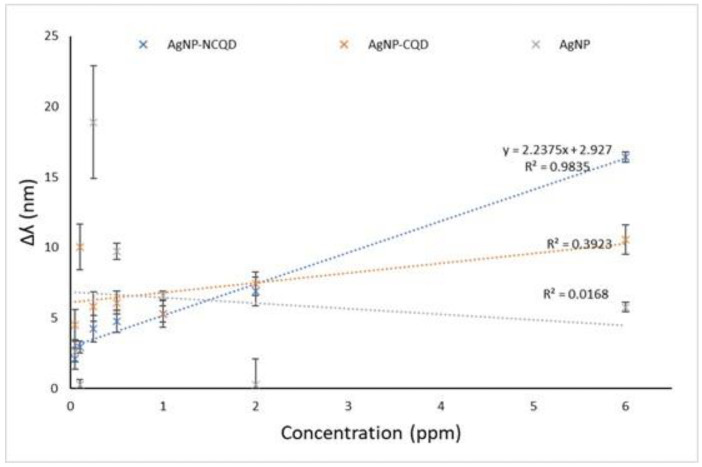
The LSPR calibration curve of bare AgNPs, AgNPs-CQD, and AgNPs-NCQD.

Based on the finding above, the calibration curve was established by plotting the wavelength shift versus chlorophyll concentration within 0.05 to 6 ppm (Figure 6). The repeatability experiment of all the sensor substrates was carried out. The AgNPs, AgNPs-CQD, and AgNPs-NCQD substrates were used to measure the various concentration of chlorophyll solution five times. The calculated correlation coefficients calibration line of AgNPs-NCQD was equal to R^2^ = 0.9835, while the calibration equation was Δ*λ* = 2.2375[chlorophyll] + 2.927. Compared to all sensing materials, AgNPs-NCQD exhibited the correlation coefficient higher compared to AgNPs (R^2^ = 0.0168) and AgNPs-CQD (R^2^ = 0.3923). The limit of detection (LOD = 3 sb/m) of the LSPR technique was calculated to be 1.02, 12.31, and 79.21 ppm for AgNPs-NCQD, AgNPs-CQD, and AgNP, respectively. The slope of the calibration curve demonstrated the sensitivity of the optical sensor and showed that the AgNPs-NCQD had the highest sensitivity compared to AgNPs and AgNPs-CQD. In summary, Table 1 compares all the proposed sensor performances.

**Figure 7:**
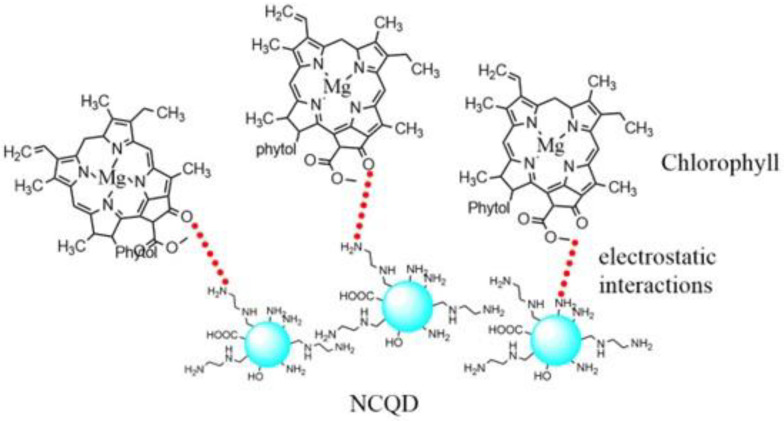
Proposed chemical interaction scheme between NCQD with chlorophyll.

Figure 7 shows the proposed interaction scheme between NCQD and chlorophyll. The result indicated interactions of surface functional groups between pristine CQD and NCQD with chlorophyll. According to the experiment results, this work has better sensitivity than reported for non-functionalized CQD [19]. In this work, the AgNPs-NCQD were functionalized with large amine groups of PEI at their surfaces. The existence of N atoms in the PEI structure plays an essential role as a chain linked to the surface of NCQD [19]. Therefore, the addition of amine groups (-NH) on the surface of NCQD led to more electrostatic interactions between -NH of NCQD and oxygen groups of chlorophyll. This paper presents the enhancement to sensitivity sensor design that detects chlorophyll concentrations in different ranges.

### 3.3. Selectivity

**Figure 8:**
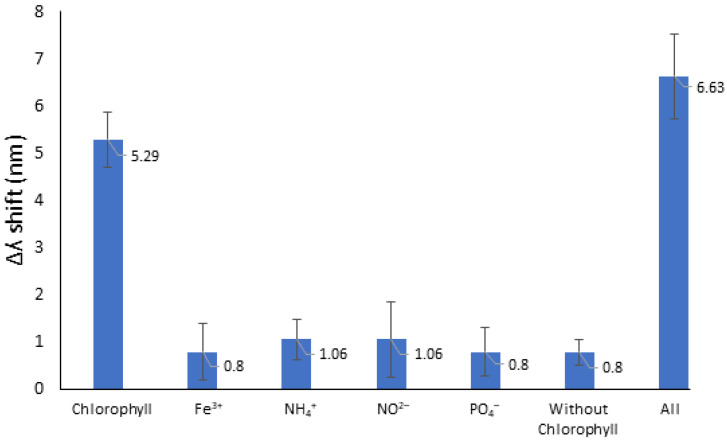
Selectivity test of the assay toward chlorophyll including NO_2_^−^, PO_4_^−^, NH_4_^+^, and Fe^3+^.

To assess the specificity of this AgNPs-NCQD film, interferences of anion and cations, including NO_2_^−^, PO_4_^−^, NH_4_^+^, and Fe_3_^+^, were investigated at the concentration of 2 ppm. As shown in Figure 8, AgNPs-NCQD LSPR-based sensor has a higher specificity for detecting chlorophyll than other interferents. Furthermore, a mixed solution that includes all interfering ions with and without chlorophyll was evaluated to investigate the anti-interference competence of the sensor. Fortunately, no significant shifted wavelength was observed despite interfering ions.

Fluorometers identify chlorophyll by shining a beam of light of a specific wavelength into the water and then detecting the higher wavelength light that is emitted. However, some handheld fluorometers inaccurately determine the chlorophyll concentrations at higher algal conditions and lower inorganic turbidity levels [27]. Therefore, the present sensor technique in this report is susceptible to the detection range (0.05–6 ppm). As shown in Table 2, the test results obtained using the sensor were compared following the known methods. CQD mainly focused on fluorescence-based sensing. The proposed sensor indicates that the carbon nano-based materials can be integrated with a medium for in situ measurement of chlorophyll, such as optical fibre, to evaluate the state of water bodies’ quality. Optical fibre offers much attention in sensor applications owing to its real-time, high sensitivity, and rapid sensing technology. In addition, integrating the sensing material as a transducer layer on the optical fibre improves the achievement of selective and sensitive detection for specific target molecules. According to the work by Yap et al., chemical modification of nanomaterials CQD as sensing elements on the tapered optical fibre offers great sensitivity and practicability for onsite detection for sensing application [28].

## 4. Conclusions

In summary, this report demonstrated an AgNPs-NCQD film-based LSPR sensor for chlorophyll detection. The performance of the sensor layer has been investigated and compared with AgNPs and AgNPs-CQD by observing the wavelength shift of reflectance spectra. The additions of amino groups to the CQD provide more active sites for chlorophyll attachment, resulting in increased sensor capability. Chlorophyll reacts with the amine groups of NCQD to form electrostatic interactions. The result shows that AgNPs-NCQD film positively responded to chlorophyll concentration with a sensitivity of 2.23 nm ppm^−1^ in the range of 0.05 to 6 ppm. The LOD and LOQ were calculated to be 1.03 ppm and 3.40 ppm, respectively. The AgNPs-NCQD also displays high interference performance against coexisting ions. It has been noticed that the AgNPs-NCQD film based-LSPR sensor had high selectivity and sensitivity, as well as an excellent linear relationship (R^2^ = 0.9835) compared to AgNPs and AgNPs-CQD. This approach provides a potential real-time detection method for chlorophyll by incorporating AgNPs-NCQD films on optical fibres to achieve easy and fast detection for analysts in the environmental biological fields. Therefore, in future work, the focus will be on preparing sensing probes and the performance optimization of fibre optic sensors.

## Figures and Tables

**Table 1:** The performance and correlation coefficient R2 of AgNPs, AgNPs-CQD, and an AgNPs–NCQD composite for chlorophyll detection.

Compound Film	R^2^	Sensitivity (nm ppm^−1^)	Range (ppm)	LOD (ppm)	LOQ (ppm)
AgNPs	0.0168	0.40	0.05-6	79.21	264.05
AgNPs-CQD	0.3923	0.70	0.05-6	12.31	41.04
AgNPs-NCQD	0.9835	2.23	0.05-6	1.02	3.40

**Table 2:** Comparison of chlorophyll detections using various methods.

Methods	Analytes	Sensitivity	Range (ppm)	LOD (ppm)	References
Fluorescence	Green algae	-	-	1	[29]
Fluorescence	Chlorophyll-a	-	-	0.001	[30]
Fluorescence	Chlorophyll-a	-	0.025–0.15	-	[27]
Fluorescence	Chlorophyll-a	1 mV 2.5 μg L^−1^	0–0.2	-	[31]
LSPR	Chlorophyll	0.80 nm ppm^−1^	0.2–10	4.71	[19]
LSPR	Chlorophyll	2.23 nm ppm^−1^	0.05–6	1.02	This work

## Data Availability

Not applicable.
